# Oxidized Low-Density Lipoprotein Promotes *In Vitro* Calcification

**DOI:** 10.3390/ma13225120

**Published:** 2020-11-13

**Authors:** Mamiko Yamashita, Yoshiaki Nomura, Misao Ishikawa, Shinji Shimoda, Nobuhiro Hanada

**Affiliations:** 1 Department of Translational Research, Tsurumi University School of Dental Medicine, Yokohama 230-8501, Japan; yamashita-m@tsurumi-u.ac.jp (M.Y.); hanada-n@tsurumi-u.ac.jp (N.H.); 2 Department of Oral Anatomy, Tsurumi University School of Dental Medicine, Yokohama 230-8501, Japan; ishikawa-misao@tsurumi-u.ac.jp (M.I.); shimoda-s@tsurumi-u.ac.jp (S.S.)

**Keywords:** oxidized low-density lipoprotein, amorphous calcium phosphate, calcification, crystallization, dentin

## Abstract

Calcification plays an important role in the human body in maintaining homeostasis. In the human body, the presence of a high amount of oxidized low-density lipoprotein (ox-LDL) is a consistent feature of the local areas that are common sites of ectopic calcification, namely dental calculus, renal calculus, and the areas affected by arteriosclerosis. Hence, ox-LDL may have some effect on calcification. Scanning electron microscopy (SEM) observation revealed a high amount of amorphous calcium phosphate (ACP) when ox-LDL was included in the solution. In the in vitro experiment, the highest amount of precipitation of calcium phosphate was observed in the solution containing ox-LDL compared to the inclusion of other biomaterials and was 4.2 times higher than that of deionized water for 4.86 mM calcium and 2.71 mM phosphate. The morphology of calcium phosphate precipitates in the solution containing ox-LDL differed from that of the precipitates in solutions containing other biomaterials, as determined by transmission electron microscopy (TEM). Through the time course observation of the sediments using TEM, it was observed that the sediments changed from spherical or oval shape to a thin film shape. These results indicate that sediments acquired a long-range order array, and the phase transitioned from non-crystalline to crystalline with an increased time and density of ACP. Thus, it is concluded that ox-LDL promoted ACP precipitation and it plays an important role in ectopic calcification.

## 1. Introduction

Intrinsic calcification is observed in the bones and teeth, while ectopic calcification occurs in the kidneys, arteries, and oral cavity [1].

Normal calcified tissue is composed of calcium phosphate, the extracellular matrix, and biologically active substances. Representative substances for calcium phosphate are apatite crystal in bone or teeth, collagen for the extracellular matrix, and bone morphogenetic protein (BMP) and dentin sialoprotein (DSP) for biologically active substances [2,3].

In addition, calcification occurs ectopically in the kidneys, arteries, and oral cavity and is known as renal calculus, arteriosclerosis, and dental calculus, respectively. This is defined as calcium sedimentation occurring in areas other than intrinsic tissue in the human body, and factors other than BMP or DSP may be involved in calcification. Previously, nanobacteria-like particles, which are known to be associated with ectopic calcification, were purified, and their components were found to consist of oxidized acidic lipids. In addition, oxidized low-density lipoprotein (ox-LDL) is a well known causative agent of arteriosclerosis [4,5,6,7].

Lipids are readily oxidized. Macrophages have been changed to foam cells by ox-LDL [8] where aggravated arteriosclerosis tissue as well as a large accumulation of lipids and foam cells were observed [9]. It was reported that the gingival crevicular fluid (GCF) of patients with a periodontal disease contained a large amount of ox-LDL, and the ox-LDL/LDL ratio was 17 times higher than that of plasma [10]. Dental calculus is often detected in gingival sulcus and the tooth surface of patients with periodontal disease. Dental calculous formation is a dynamic process of mineralization and demineralization. In GCF, Fetuin, which has an inhibitory effect of calcification, were contained [11]. Ox-LDL may act in the ectopic calcification of the oral cavity.

This evidence suggests that ox-LDL may play an important role in calculus formation [10,12] though no mechanism has yet been elucidated. In addition, there exists a variety of substances in blood, GCF, and tissue fluid, and there is a need to compare the calcification ability of ox-LDL in the presence of these substances.

Ox-LDL is known to be an etiological substance of non-communicable diseases [13,14]. However, ox-LDL is also a physiologically active substance in intrinsic calcification. The tooth surface is always exposed to acids produced by oral commensal bacteria. Dental caries is a process controlled by the balance of mineralization and demineralization. Ox-LDL may act as a promoting agent in the calcification of teeth and bone.

The aim of this study was to confirm the effect of ox-LDL on calcification by an in vitro and in situ study.

## 2. Materials and Methods

### 2.1. Effect of Biomaterials on Calcium Phosphate Sedimentation

#### 2.1.1. Experimental Procedure

Calcium phosphate sedimentation was evaluated by the turbidity measured by spectrophotometer (UV-1200, SHIMADZU, Kyoto, Japan), with a wavelength of 650 nm. A 2.67 μL aliquot of calcium and phosphate solution (Ca/P = 1.79) [15,16,17] was added dropwise to 800 μL of biomaterial solution in deionized water. The calcium phosphate solution was composed of 732.6 mM Ca(NO_3_)_2_∙4H_2_O and 408.2 mM (NH_4_)_2_HPO_4_. A total of 25 stepwise drops were performed. In one experiment, 2.44 mM Ca (NO_3_)_2_∙4H_2_O and 1.36 mM (NH_4_)_2_HPO_4_ were added up to a final concentration of 56.42 mM for Ca^2+^ and 31.44 mM for PO_4_^−^.

The biomaterials used in this study are as follows:

Protein—albumin (from bovine serum, Fraction V, pH = 7.0, FUJIFILM Wako Pure Chemical Corporation, Osaka, Japan).

Fats, unsaturated fatty acid-docosahexaenoic acid (DHA) (4,7,10,13,16,19-docosahexaenoic acid, Molecular weight (MW) = 328.50, Nakarai Tesque, Kyoto, Japan), eicosapentaenoic acid (EPA) (5,8,11,14,17-eicosapentaenoic acid, MW = 302.46, Nakarai Tesque, Kyoto, Japan).

Carbohydrates—dextran sulfate (dextran sulfate sodium salt, MW36,000–50,000, FUJIFILM Wako Pure Chemical Corporation, Osaka, Japan) for anionic charged biomaterial, and polyethyleneimine (average MW = 10,000, FUJIFILM Wako Pure Chemical Corporation, Osaka, Japan) for cationic charged molecules.

Glycolipids—lipopolysaccharides from *Escherichia coli* (FUJIFILM Wako Pure Chemical Corporation, Osaka, Japan) and from *Porphyromonas gingivalis* (LPS-PG: LPS from *P. gingivalis*, Thermo Fisher Scientific, Tokyo, Japan).

Lipoprotein—high-density lipoprotein (HDL, human, Thermo Fisher Scientific, Tokyo, Japan), low-density lipoprotein (LDL, human, Thermo Fisher Scientific, Tokyo, Japan), oxidized low-density lipoprotein (ox-LDL, human, Thermo Fisher Scientific, Tokyo, Japan).

The concentrations of these solutions were each 0.025% (w/w). To evaluate the effects of concentrations, 0.1% solutions were also used. The ox-LDL and dextran sulfate experiments were independently repeated three times.

#### 2.1.2. Statistical Analysis

Two-way analysis of variance (two-way ANOVA) was applied for the effect of biomaterials and the time for the absorbance by following formula. Absorbance was used as the dependent variable, and calcium phosphate concentration, biomaterials and the interaction of biomaterials and calcium phosphate concentrations were used as the dependent variables. Deionized water was used as a reference.

Analysis was performed by SPSS ver 24.0 (IBM, Tokyo, Japan):

(1) $$\begin{array}{cc} {P(\mathrm{Absorvance})}_{\mathrm{im}} & \\ & ={\left(\mathrm{Calcium}\mathrm{phosphate}\right)}_{i}\\ & +\sum_{m=1}^{10}\beta_{m}{\left(\mathrm{Biomaterials}\mathrm{indexed}\mathrm{by}m\right)}_{\mathrm{im}}\\ & +\sum_{m=1}^{10}{\left(\mathrm{Biomaterials}\mathrm{indexed}\mathrm{by}m\right)}_{\mathrm{im}}\\ & \times {\left(\mathrm{Biomaterials}\mathrm{indexed}\mathrm{by}m\right)}_{\mathrm{im}}+{\mathrm{Intercept}}_{\mathrm{im}}+e_{\mathrm{im}} \end{array}$$

(2) $$\mathrm{where}\;\mathrm{absorbance}\;\sim N\left(0,\delta_{e}^{2}\right),\;e_{im}\sim N\left(0,\delta_{e}^{2}\right)$$

### 2.2. Solid-Phase Analysis

Precipitation was observed by transmission electron microscopy (TEM) and scanning electron microscopy (SEM). Calcium phosphate molar ratio (Ca/P) was calculated using an electron probe micro-analyzer (EPMA).

Samples were precipitated using low and high concentrations of calcium and phosphate: 9.65 mM calcium and 5.38 mM phosphate for the low concentration and 32.70 mM calcium and 18.22 mM phosphate for the high concentration. The analysis of time-dependent changes for the low concentration were performed at 5, 30 min, and 24 h after the addition of the calcium phosphate solution.

#### 2.2.1. Transmission Electron Microscopy Observation

Each precipitate was centrifuged at 6000 rpm for 1 min, and the precipitates were washed with 100% ethanol 3 times to stop the reactions. Samples were dropped on a collodion-coated EM grid. Observations were carried out by TEM (JEM-1200CX, JEOL, Tokyo, Japan) under an acceleration voltage of 80 kV, and the electron diffraction pattern was obtained with the camera length set at 80 cm. Monoclinic hydroxyapatite (FUJIFILM Wako Pure Chemical Corporation, Osaka, Japan) was used as a control.

#### 2.2.2. Scanning Electron Microscopy Observation

All samples for the SEM observation were dehydrated by serial ethanol alcohol, and then freeze-dried with t-butyl alcohol. The samples were gold ion-coated after being freeze-dried. SEM observation was carried out (JCM-6000 JEOL, Tokyo, Japan) under an acceleration voltage of 15 kV.

#### 2.2.3. Analysis of the Calcium Phosphate Molar Ratio with Electron Probe Micro-Analyzer (EPMA)

Precipitate samples were subjected to the quantitative line analysis of calcium and phosphate (wavelength dispersion system) using an EPMA (JXA9200Ⅱ, JEOL, Tokyo, Japan). The analysis conditions were as follows: an accelerating voltage of 20 kV and a current value of 5 × 10^−8^ A. Under the same conditions as the line analysis of the sample, line analysis was performed to determine the calcium phosphate molar ratio (Ca/P) of four chemicals. Then, a calibration curve was drawn using the Ca/P value of the chemicals and the calcium and phosphate counts (CPS) obtained by the analysis. From this calibration curve, the Ca/P ratio was determined from the CPS of the sample.

The used chemicals are as follows:

HAP (hydroxyapatite)—Ca_10_(PO_4_)_6_(OH)_2_, Ca/P = 1.67 (FUJIFILM Wako Pure Chemical Corporation, Osaka, Japan);

DCPA (brushite, monetite)—CaHPO_4_, Ca/P = 1.0 (FUJIFILM Wako Pure Chemical Corporation, Osaka, Japan);

TCP (tricalcium phosphate)—Ca_3_(PO_4_)_2_, Ca/P = 1.5 (FUJIFILM Wako Pure Chemical Corporation, Osaka, Japan);

TTCP (tetracalcium phosphate, monoclinic)—Ca_4_(PO_4_)_2_O, Ca/P = 2.0 (FUJIFILM Wako Pure Chemical Corporation, Osaka, Japan).

### 2.3. Dentin Mineralization Experiments

#### 2.3.1. Dentin Sample Preparation

First, the root of the bovine incisor was cut horizontally to the vertical axis of the tooth. Then, for mineralization experiments, samples (3 mm × 1 mm × 1 mm) of the root surface was prepared from that part after mechanical removal of periodontal ligaments and debris. Samples were also demineralized by immersion in 10% formic acid solutions for 24 h. Samples were transected for the dentinal tubules, then immersed in 0.05% NaOCl for 5 min.

These samples were washed with distilled water twice for 5 min, then dehydration was performed stepwise by applying increasing ethanol concentrations. Samples were freeze-dried with t-butyl alcohol.

#### 2.3.2. Mineralization of Root Surface

For the mineralization experiments, demineralized samples were immersed in 800 μL of 0.025% ox-LDL or distilled water for the 37.38 μL calcium and phosphate solution (Ca = 32.7 mM and P = 18.22 mM) and mixed into the solutions. The samples were then incubated at 37 °C for 24 h.

These samples were prepared for the observation for SEM using the same procedures described in Section 2.2.

## 3. Results

### 3.1. Effect of Biomaterials on the Calcium Phosphate Crystallization

#### 3.1.1. Effect of ox-LDL on the Mineralization on the Dentin Surface

**Figure 1:**
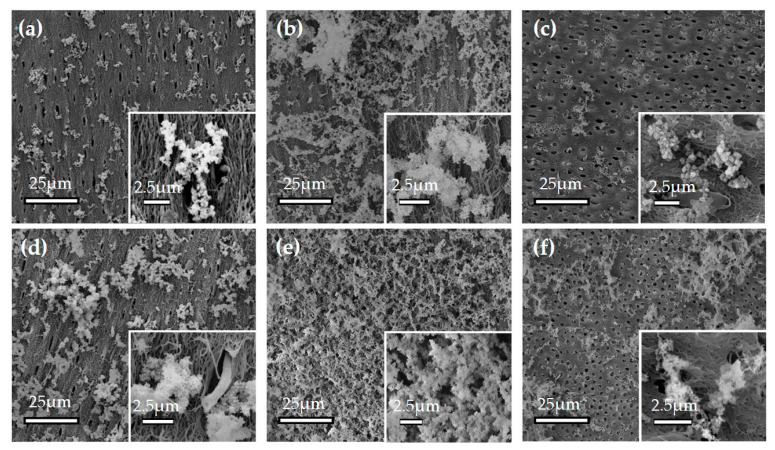
SEM image of the calcium phosphate precipitate with biomaterials. (**a**) Precipitate from 4.86 mM calcium and 2.71 mM phosphate without biomaterials; (**b**) 4.86 mM calcium and 2.71 mM phosphate with 0.025% oxidized low-density lipoprotein (ox-LDL); (**c**) 4.86 mM calcium and 2.71 mM phosphate with 0.025% dextran sulfate; (**d**) 23.66 mM calcium and 13.18 mM phosphate without biomaterials; (**e**) 23.66 mM calcium and 13.18 mM phosphate with 0.025% ox-LDL; and (**f**): 23.66 mM calcium and 13.18 mM phosphate with 0.025% dextran sulfate.

Crude dentin may be affected by the elution of minerals contained in dentin, so demineralized dentin obtained by the elimination of apatite crystals was used. The SEM observation of the sample before mineralization experiments is shown in Figure S1. Dentin apatite crystals were removed, and collagen fibers that surrounded dentin tubules were observed. The effects of calcium and phosphate concentrations were compared by using 4.86 mM calcium and 2.71 mM phosphate (Figure 1a–c) and 23.66 mM calcium and 13.18 mM phosphate (Figure 1d–f).

The samples in Figure 1a,d were immersed in the calcium phosphate solutions without biomaterials while those in b and e were with ox-LDL and those in Figure 1c,f were with dextran sulfate.

A high amount of precipitate was observed in the ox-LDL-containing solutions (Figure 1b,e). For all samples, a higher amount of precipitate was observed with the concentration of 23.66 mM calcium and 13.18 mM phosphate (Figure 1d–f).

#### 3.1.2. Ca/P Analysis of Precipitates with EPMA

The calcium and phosphate ratios of the precipitates were analyzed for the 39.3 mM calcium and 21.9 mM phosphate solution containing 0.025% ox-LDL. Deionized water was used as a control. In the line analysis, the calcium and phosphate ratios were 1.28 for the ox-LDL-containing solution and 1.06 for the control.

#### 3.1.3. Effects of Calcium and Phosphate Concentrations

**Figure 2:**
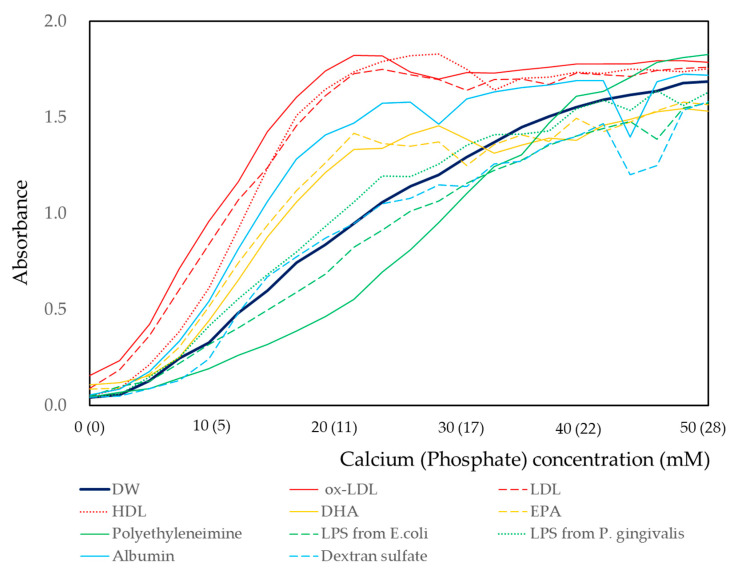
Effect of the biomaterials on calcium and phosphate reaction. Absorbance values were measured at a wavelength of 650 nm. DW: Deionized water, LDL: Low-density lipoprotein, HDL: high-density lipoprotein, DHA: Docosahexaenoic acid, EPA: Eicosapentaenoic acid, LPS: lipopolysaccharides.

The effect of biomaterials on the calcium and phosphate reaction was measured by turbidity using a spectrophotometer. The concentration of biomaterials was fixed at 0.025%, and the calcium and phosphate concentrations were increased in a stepwise manner using 25 intervals (Figure 2). Deionized water without biomaterials was used as the control. For the deionized water, the increase in absorbance was gentle until the concentration reached less than 4.86 mM calcium and 2.71 mM phosphate. Then, a linear increase in absorbance was observed until the concentration of 30.46 mM calcium and 16.97 mM phosphate. The increases in absorbance then gradually flattened and reached a plateau. The increases in absorbance resembled a sigmoid curve. When ox-LDL, LDL, or HDL was included in the solutions, steep increases in absorbance were observed from low concentration (4.86 mM calcium and 2.71 mM phosphate). The concentrations corresponding to an absorbance peak were 21.36 mM calcium and 11.90 mM phosphate for ox-LDL, 23.66 mM calcium and 13.18 mM phosphate for LDL, and 28.21 mM calcium and 15.72 mM phosphate for HDL. Then, the curves fluctuated and, finally, these curves plateaued at high concentrations. When albumin, EPA, or DHA was included in the solutions, the curves were located between those of ox-LDL and deionized water. Curves of dextran sulfate or LPS from *E. coli* or *P. gingivalis* were similar to that of deionized water. The lowest increase in absorbance was observed for the solution containing polyethyleneimine. The increases in absorbance all resembled sigmoid curves and plateaued at 32.70 mM calcium and 18.22 mM phosphate. In all cases, the turbidity increased with the increase in calcium and phosphate concentrations. The physiological calcium concentration in the human body is known to be around 5 mM. At this concentration, the relative absorbance of biomaterial solutions is calculated by dividing by that of deionized water. The result is shown in Figure S2. The absorbance of the ox-LDL-containing solution was 4.2 times that of deionized water. The effect of biomaterials on the calcium and phosphate reactions was analyzed by two-way analysis of variance. The results are shown in Table 1. Deionized water was used as a reference. The coefficient of ox-LDL was the highest, that of polyethyleneimine was negative, and those of LPS and dextran sulfate were not statistically significant.

#### 3.1.4. Effect of Concentration of Biomaterials on Calcium and Phosphate Reaction

**Figure 3:**
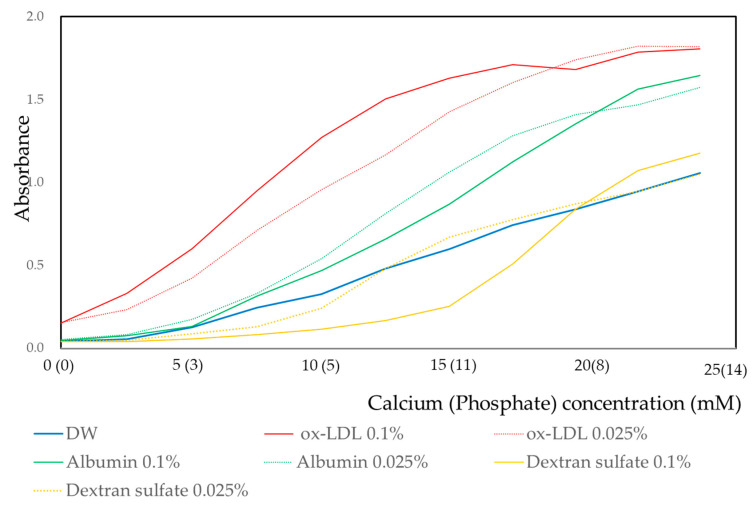
Effect of the concentration of biomaterials on the calcium and phosphate reaction. Absorbance values were measured at a wavelength of 650 nm.

For ox-LDL, albumin, and dextran sulfate, the effect of concentrations of these biomaterials was investigated using 0.025% and 0.1% concentrations. The results are shown in Figure 3. For ox-LDL, the sigmoid curve was shifted upward for the 0.1% concentration. However, for albumin and dextran sulfate, sigmoid curves were shifted downward for the 0.1% concentration.

### 3.2. Solid-Phase Analysis of Precipitates by Electron Microscopy

#### 3.2.1. Transmission Electron Microscopy Analysis

##### Effect of Biomaterials on the Morphology of Precipitates as Determined by TEM Observation

**Figure 4:**
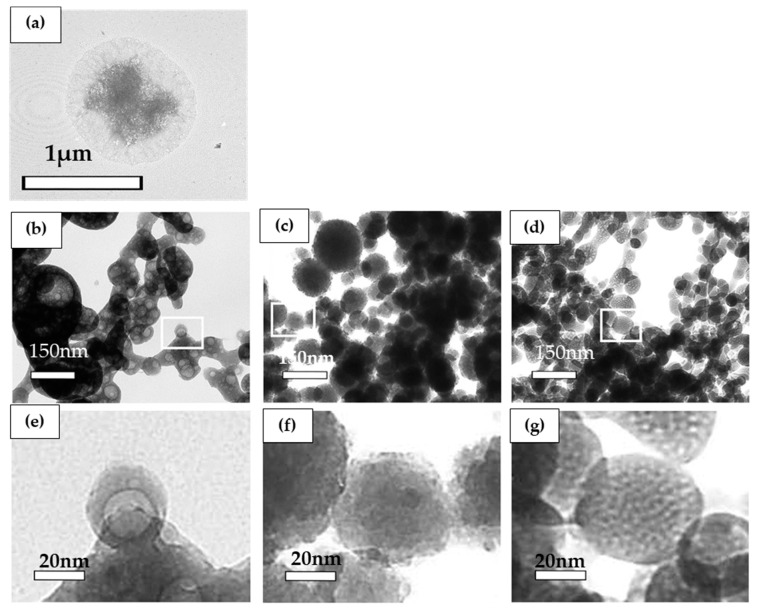
Observation of calcium phosphate precipitation by transmission electron microscopy. Calcium and phosphate concentrations were 12.02 mM and 6.70 mM. The concentrations of biomaterials were 0.025%. (**e**)–(**g**) are high magnifications of the white squares in (**b**)–(**d**); (**a**) without biomaterials; (**b**) oxidized low-density lipoprotein (ox-LDL); (**c**) albumin,; (**d**) dextran sulfate; (**e**) ox-LDL; (**f**) albumin; (**g**) dextran sulfate. Magnifications: (**a**) 10,000× (**b**)–(**g**): 30,000×.

Precipitation in solution at the concentration of 12.02 mM calcium and 6.70 mM phosphate with or without biomaterials was observed by transmission electron microscopy (TEM). For the control without biomaterial, high electron density areas surrounded by a low-density area were observed (Figure 4a). By contrast, for the solution with 0.025% ox-LDL, low electron density areas surrounded by a high-density area were observed (Figure 4b,e).

For the solution containing albumin and dextran sulfate, high-density spherical precipitates were observed (Figure 4c,d). Under observation at high magnification, small protrusions were observed for the precipitates in the solution containing albumin (Figure 4c,f). For the solution containing dextran sulfate, the surfaces of the spherical precipitates were smooth. For the solution containing dextran sulfate, low-density precipitates were scattered in high-density precipitates (Figure 4d,g).

##### The Effect of Calcium and Phosphate Concentrations on the Ox-LDL-Containing Precipitates as Determined by Electron Microscopy

**Figure 5:**
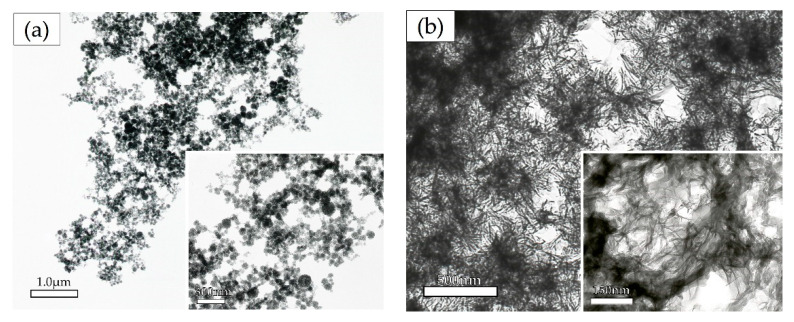
TEM images of the precipitate with 0.025% ox-LDL: (**a**) 12.02 mM calcium and 6.70 mM phosphate; (**b**) 39.33 mM calcium and 21.92 mM phosphate.

The crystal shapes with 0.025% ox-LDL obtained by high and low concentrations were observed using transmission electron microscopy (TEM). For the low concentration, 12.02 mM calcium and 6.70 mM phosphate, spherical or oval high electron density precipitates were observed (Figure 5a). For the high concentration, 39.33 mM calcium and 21.92 mM phosphate, mesh-shaped high electron density crystals were observed (Figure 5b). These high-density mesh-shaped precipitates were clearly different from the low concentration oval-shaped precipitates.

##### Time Course Analysis of the Crystal Shape in the Solution Containing 0.025% Ox-LDL

**Figure 6:**
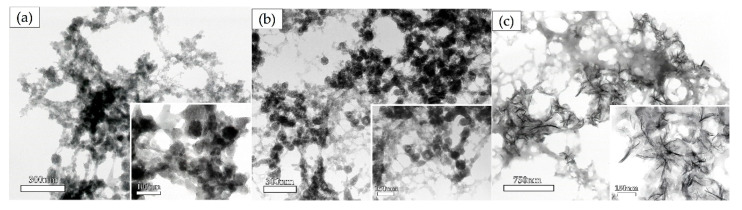
Time course analysis of the crystal shape in the solution containing 0.025% ox-LDL by transmission electron microscopy: (**a**) 5 min; (**b**) 30 min; (**c**) 24 h.

For this analysis, 3.00 mM calcium and 1.68 mM phosphate were included in the solution containing 0.025% ox-LDL. After 5, 30 min, and 24 h, the precipitates were observed by TEM (Figure 6). For the 5 min reaction, spherical or oval-shaped precipitates were observed (Figure 6a). For the 30 min reaction, membrane-shaped precipitates appeared in between spherical or oval-shaped precipitates (Figure 6b). For the 24 h reaction, mesh-shaped precipitates became major components, and the aggregation of needle-shaped precipitates was observed (Figure 6c). These needle-shaped precipitates were visibly different from those of the 5 min reaction, namely the spherical or oval-shaped precipitates. The 24 h reaction by albumin and dextran is shown in Figure S3. The capacity of the aggregation of calcium and phosphate was lower than ox-LDL. High electron density areas were observed for ox-LDL. In contrast, the layers were thinner for albumin and dextran sulfate.

##### Identification of the Precipitates by Selected Area Electron Diffraction Analysis

To identify the precipitates, selected area electron diffraction analysis was carried out. The result is shown in Figure S3. The diffraction pattern obtained for the precipitates showed a halo pattern, such as that for amorphous calcium phosphate (ACP; Figure S4b), and these halo patterns were also obtained for rounded or oval-shaped precipitates. Regarding the other precipitates, patterns for known samples such as hydroxyapatite were obtained, which showed a spotted diffraction pattern (Figure S4a).

## 4. Discussion

In this study, ox-LDL was observed to have a promoting effect on calcification in the in vitro and in situ experiments. Concentrations of calcium and phosphate in the human body are regulated by homeostasis. Imbalance in calcium concentrations leads to ectopic calcification which, in turn, leads to the development of non-communicable diseases [1].

Ectopic calcification is mostly crystalline, with a pattern consisting of an array of three-dimensionally repeated units [10,12,18,19]. In contrast, non-crystalline calcification has an array of a short-range order. An array with a short-range order has been observed for amorphous calcium phosphate (ACP) [20,21,22]. In this study, an at least 20 mM concentration of calcium is necessary. The serum level of calcium concentration is known to be from about 5 to 10 mM. In fact, there exist dental calculus and renal calculus in human body with a low calcium concentration in serum. Under the conditions of the formations these substances, a calcium concentration of at least more than 20 mM is necessary.

The observation of ACP under SEM shows a spherical shape. The Ca/P of ACP was 1.0 to 1.5, and it was higher than that of calcium phosphate. Arrays of long-range order, like crystalline calcification, were not observed [23,24]. The sediments obtained in this study were spherical or oval-shaped with φ50–150 nm (Figure 1, Figure 2, Figure 3, Figure 4, Figure 5 and Figure 6). The Ca/P of the sediments obtained when including ox-LDL was 1.28. As observed in the electron diffraction analysis, the diffraction pattern showed a halo pattern, which is typical for non-crystalline calcification (Figure S3). These results indicate that the sediments obtained in this study were non-crystalline, rather than crystalline, ACP. Previous reports have shown that the phase transition of non-crystalline ACP take places in a gel and is a starting point for the production of crystalline calcium phosphate [18,19,25,26,27,28,29,30].

As shown in Figure 2 and Table 1, the highest amount of sediments was obtained in the solution with ox-LDL. Ox-LDL is an organic complex molecule consisting of oxidized lipids and partially oxidized protein. The negative charge of ox-LDL is increased through oxidation by reactive oxygen. Calcium ions bind to the negatively charged region of ox-LDL [31,32]. This may be the cause of the high amount of sedimentation in the solution containing ox-LDL. However, when compared to the results for dextran and dextran sulfate, the amount of sedimentation was larger in the solution containing dextran. The electronegativity of the molecule is not the only cause of the increased amount of sedimentation. In the ox-LDL molecule, deletions often occur as a result of oxidation. This leads to the increase in surface area of the ox-LDL molecule. Calcium and phosphate ions may aggregate in the ox-LDL molecule. This may be one of the reasons for increased sedimentation in the solution containing ox-LDL.

The precipitation of crystalline calcium phosphate is dependent on temperature, pressure, and pH. Hydroxyapatite (HAP) precipitation is observed at concentrations of 10^−8^ to 10^−9^ M under the conditions of 37 °C, 1 Pa, and pH 7.0 [33,34]. If the ox-LDL induces the phase transition for ACP to other crystalline forms of calcium phosphate, like HAP, dicalcium phosphate (DCP), tricalcium phosphate (TCP), or octacalcium phosphate (OCP), and the amount of sediments may increase with the increasing ox-LDL concentration. As shown in Figure 3, the amount of sedimentation was higher in the solution containing 0.1% ox-LDL than in the solution containing 0.025% ox-LDL. In contrast, in the solutions containing albumin or dextran sulfate, a higher amount of sedimentation was observed in 0.025% than in 0.1%. In the solution containing ox-LDL, morphological changes in sediment shape from spherical or oval to thin film are dependent on the concentrations of calcium phosphate and reaction time (Figure 5 and Figure 6). These morphological changes indicate the transition from an irregular to a regular array of atoms. The sediments acquired an array of long-range order repeated atoms. This indicates that the phase transitioned from non-crystalline to crystalline. Ox-LDL may have the Ca/P activity to promote the formation of ACP and phase transformation from non-crystalline to crystalline.

High molecular weight proteins have an inhibitory effect on the crystal growth of calcium phosphate. Calcium phosphate crystals are surrounded by high molecular weight protein and are degraded by the enzymatic reactions. It has been reported that the high molecular weight proteins surrounding the crystals are reduced in molecular weight by enzymatic action to promote the growth of crystals [35]. As shown in Figure 4a, sediments without biomaterials had a high electron density area due to calcium phosphate in the center of crystals which was surrounded by a low electron density area due to ammonium and nitric acid. Sediments in the solution containing albumin had small protrusions, and low electron density areas were observed (Figure 4c,f). These results are consistent with a previous report [35]. By contrast, sediments in the solution containing ox-LDL had a low electron density area in the center of the crystals surrounded by a high electron density area due to calcium phosphate (Figure 4b,e). The morphology of the sediments was completely different from that of albumin. The results indicate that high molecular weight proteins form calcium phosphate precipitates by surrounding the sediments. (Figure 4). ACP may precipitate around ox-LDL. For the sediments containing dextran sulfate, low electron density areas were observed as small particles scattered in the oval-shaped precipitates (Figure 4d,g). This may indicate that dextran sulfate formed a gelatinous complex with calcium phosphate.

In the human body, LDL is oxidized by local inflammation. Macrophages incorporate ox-LDL by phagocytosis. Macrophages change to foam cells, and their deposits are a cause of atherosclerosis [36,37,38]. The GCF of patients with periodontal disease contains abundant ox-LDL [10]. In this study, ox-LDL induced a high amount of precipitation of ACP (Figure 2, Figure 3, Figure 4, Figure 5 and Figure 6). The local area that contained a high amount of ox-LDL induced ACP precipitation. In the human body, the presence of high amounts of ox-LDL is a consistent feature of local areas that are common sites of ectopic calcification, namely dental calculus, renal calculus, and areas affected by arteriosclerosis [6,7]. One of the limitations of this study was not investigating the biological activity. Further study is necessary to confirm the biological activity of ox-LDL with calcification.

## 5. Conclusions

Imbalance in calcium concentrations leads to ectopic calcification. Ox-LDL promoted calcification and may be involved in the initial stages of calcification. In the human body, the presence of high amounts of ox-LDL is a consistent feature of local areas that are common sites of ectopic calcification.

## Figures and Tables

**Table 1:** Effect of the biomaterials on the reaction of calcium and phosphate.

**-**	**Coefficient (95% CI)**	***p*-Value**	-	
Calcium and Phosphate	0.138 (0.133–0.143)	<0.001		
Biomaterials				
DW	Reference			
**Biomaterials**	**Crude**		**Interaction with Calcium and Phosphate**	
	**Coefficient (95% CI)**	***p*-Value**	**Coefficient (95% CI)**	***p*-Value**
Ox-LDL	0.600 (0.519–0.680)	<0.001	−0.015 (−0.020–−0.009)	<0.001
LDL	0.497 (0.417–0.578)	<0.001	−0.015 (−0.020–−0.010)	<0.001
HDL	0.429 (0.349–0.501)	<0.001	−0.011 (−0.016–−0.006)	<0.001
DHA	0.189 (0.109–0.207)	<0.001	−0.011 (−0.016–−0.006)	<0.001
EPA	0.230 (0.150–0.311)	<0.001	−0.008 (−0.013–−0.003)	0.001
Polyethyleneimine	−0.205 (−0.286–−0.124)	<0.001	0.003 (−0.002–0.008)	0.266
LPS from *E. coli*	−0.068 (−0.148–0.013)	0 100	−0.001 (−0.006–0.004)	0.639
LPS from *P. gingivalis*	0.061 (0.020–0.142)	0.137	−0.002 (−0.007–0.003)	0.448
Albumin	0.303 (0.223–0.384)	<0.001	−0.011 (−0.016–−0.006)	<0.001
Dextran sulfate	−0.010 (−0.076–0.055)	0.756	−0.002 (−0.007–0.003)	0.354
Intercept	−0.191 (−0.253–−0.129)	<0.001	-	

The effect of biomaterials on the calcium and phosphate reactions was analyzed by two-way analysis of variance. Deionized water was used as reference. The coefficient of ox-LDL was the highest, that of polyethyleneimine was negative, and those of LPS and dextran sulfate were not statistically significant. CI: Confidence interval.

## Supplementary Materials

The following are available online at https://www.mdpi.com/1996-1944/13/22/5120/s1: Figure S1: Decalcified bovine dentin as observed by scanning electron microscopy, Figure S2: Relative absorbance of the solutions containing biomaterials, Figure S3: Crystal shape in the solution containing albumin and dextran after a 24-hour reaction, Figure S4: Selected area of the electron diffraction pattern.
