# Supplementary material for: Oxidized Low-Density Lipoprotein Promotes In Vitro Calcification
Source: Materials (Basel). 2020 Nov 13;13(22):5120. doi: 10.3390/ma13225120 (PMC7697660; doi:10.3390/ma13225120)
Supplement: Supplementary file 1 [file materials-13-05120-s001.pdf]

materials

Supplementary Materials

Oxidized Low-Density Lipoprotein Promotes *in vitro* Calcification

Mamiko Yamashita [1], Yoshiaki Nomura [1,*], Misao Ishikawa [2], Shinji Shimoda [2] and Nobuhiro Hanada [1]

[1] Department of Translational Research, Tsurumi University School of Dental Medicine, Yokohama 230-8501, Japan; yamashita-m@tsurumi-u.ac.jp (M.Y.); hanada-n@tsurumi-u.ac.jp (N.H.)

[2] Department of Oral Anatomy, Tsurumi University School of Dental Medicine, Yokohama 230-8501, Japan; ishikawa-misao@tsurumi-u.ac.jp (M.I.); shimoda-s@tsurumi-u.ac.jp (S.S.)

* Correspondence: nomura-y@tsurumi-u.ac.jp

Received: 16 October 2020; Accepted: 11 November 2020; Published: date

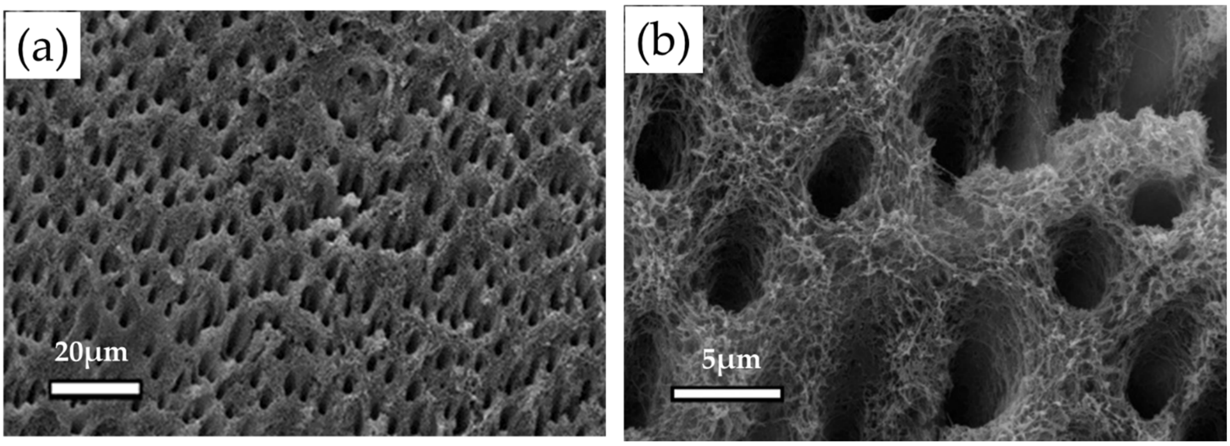

Figure 1. Decalcified bovine dentin as observed by scanning electron microscopy. (**a**) 20 μm; (**b**) 5 μm. The apatite crystals were not observed between dentin matrix collagen fibers.

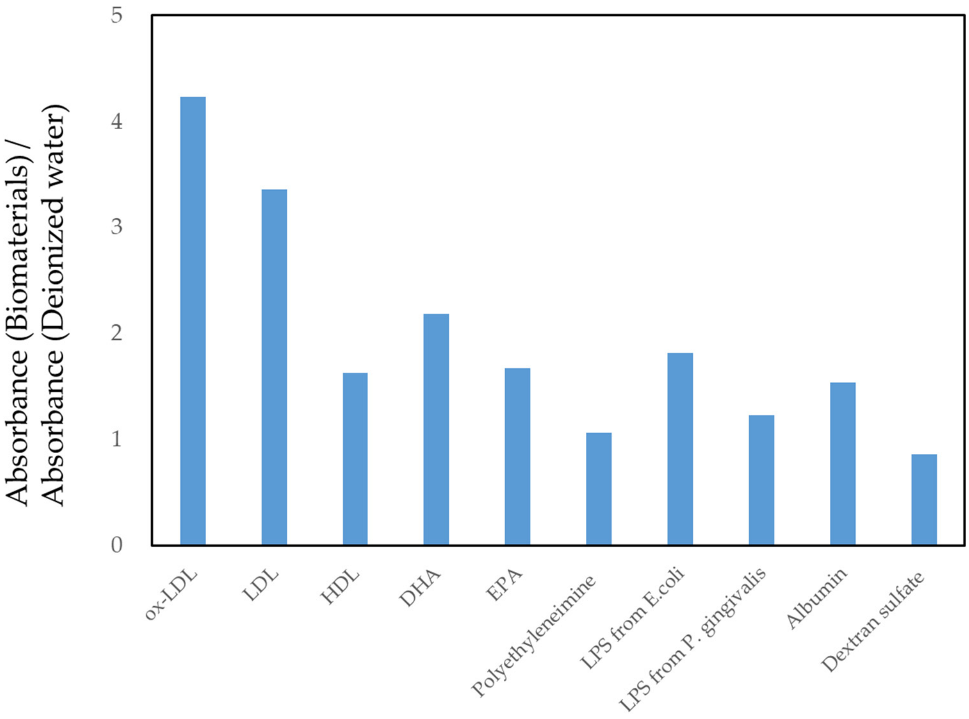

Figure S2. Relative absorbance of the solutions containing biomaterials. Absorbances of the solutions of biomaterials were divided by that of deionized water. Solution containing ox-LDL was highest and 4.2 times that of deionized water. Absorbance obtained by 4.86mM calcium, 2.71mM phosphate and 0.025% biomaterials.

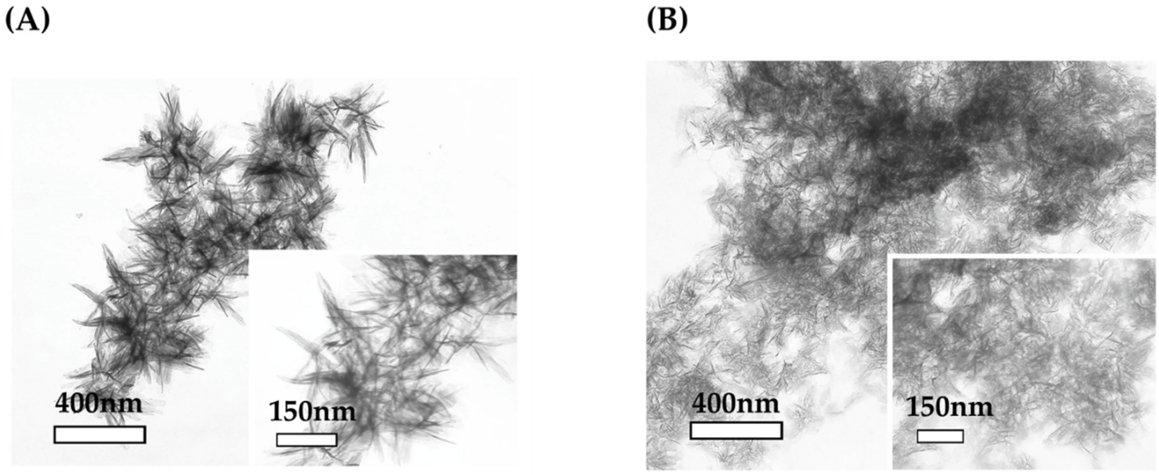

Figure S3. Crystal Shape in the Solution Containing albumin and dextran after 24 h reaction.(**A**) Albumin (**B**) Dextran sulfate.

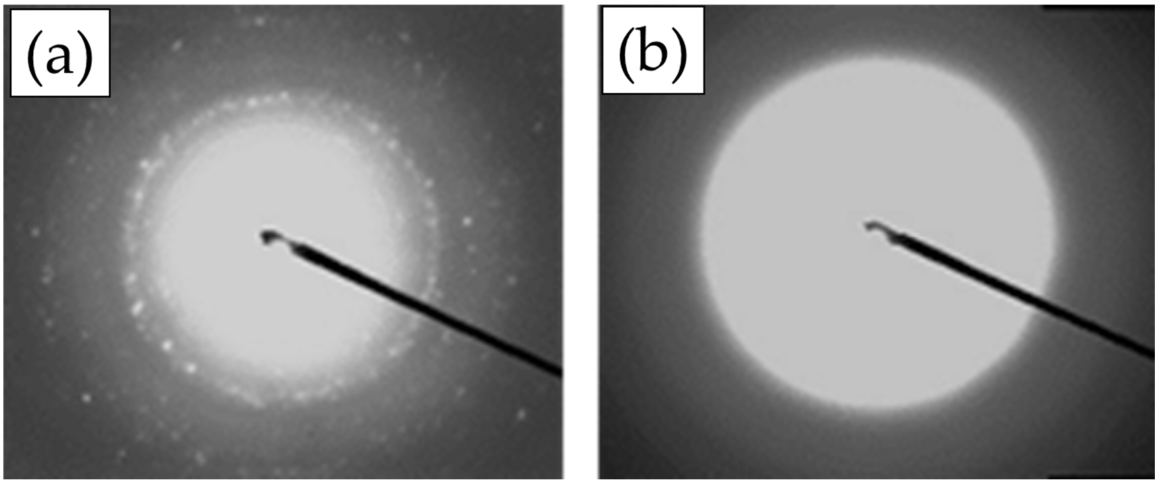

Figure 4. Selected area of electron diffraction pattern.(**a**): Diffraction spots obtained from hydroxyapatite crystal.(**b**): Halo pattern obtained from the oval shaped precipitates.

© 2020 by the authors. Submitted for possible open access publication under the terms and conditions of the Creative Commons Attribution (CC BY) license (http://creativecommons.org/licenses/by/4.0/).
